# Supplementary material for: Collection of Controlled Nanosafety Data—The CoCoN-Database, a Tool to Assess Nanomaterial Hazard
Source: Nanomaterials (Basel). 2022 Jan 28;12(3):441. doi: 10.3390/nano12030441 (PMC8839907; doi:10.3390/nano12030441)
Supplement: Supplementary file 1 [file nanomaterials-12-00441-s001.zip › Supplementary Material Reference List in vitro Studies final1.pdf]

References

3.1. In vitro Studies

The data for figures 5 A-C were extracted from the publications listed below.

3.1.1. Acute Toxicity (Fig. 5A) - [1-18]

3.1.2. ROS Formation (Fig. 5B) - [2,3,7,9,10,13,14,17-22]

3.1.3. Cytokine Production (Fig. 5C) - [1,2,13,16,19,20]

1. Dekali, S.; Divetain, A.; Kortulewski, T.; Vanbaelinghem, J.; Gamez, C.; Rogerieux, F.; Lacroix, G.; Rat, P. Cell cooperation and role of the p2x(7) receptor in pulmonary inflammation induced by nanoparticles. *Nanotoxicology* **2013**, *7*, 1302-1314.
2. Ekstrand-Hammarstrom, B.; Akfur, C.M.; Andersson, P.O.; Lejon, C.; Osterlund, L.; Bucht, A. Human primary bronchial epithelial cells respond differently to titanium dioxide nanoparticles than the lung epithelial cell lines a549 and beas-2b. *Nanotoxicology* **2012**, *6*, 623-634.
3. Frick, R.; Muller-Edenborn, B.; Schlicker, A.; Rothen-Rutishauser, B.; Raemy, D.O.; Gunther, D.; Hattendorf, B.; Stark, W.; Beck-Schimmer, B. Comparison of manganese oxide nanoparticles and manganese sulfate with regard to oxidative stress, uptake and apoptosis in alveolar epithelial cells. *Toxicology letters* **2011**, *205*, 163-172.
4. Ge, Y.; Bruno, M.; Wallace, K.; Winnik, W.; Prasad, R.Y. Proteome profiling reveals potential toxicity and detoxification pathways following exposure of beas-2b cells to engineered nanoparticle titanium dioxide. *Proteomics* **2011**, *11*, 2406-2422.
5. Ho, C.C.; Chang, H.; Tsai, H.T.; Tsai, M.H.; Yang, C.S.; Ling, Y.C.; Lin, P. Quantum dot 705, a cadmium-based nanoparticle, induces persistent inflammation and granuloma formation in the mouse lung. *Nanotoxicology* **2013**, *7*, 105-115.
6. Horie, M.; Fujita, K.; Kato, H.; Endoh, S.; Nishio, K.; Komaba, L.K.; Nakamura, A.; Miyauchi, A.; Kinugasa, S.; Hagihara, Y., *et al.* Association of the physical and chemical properties and the cytotoxicity of metal oxide nanoparticles: Metal ion release, adsorption ability and specific surface area. *Metallomics.* **2012**, *4*, 350-360.
7. Horie, M.; Nishio, K.; Fujita, K.; Kato, H.; Endoh, S.; Suzuki, M.; Nakamura, A.; Miyauchi, A.; Kinugasa, S.; Yamamoto, K., *et al.* Cellular responses by stable and uniform ultrafine titanium dioxide particles in culture-medium dispersions when secondary particle size was 100 nm or less. *Toxicology in vitro : an international journal published in association with BIBRA* **2010**, *24*, 1629-1638.
8. Hsiao, I.L.; Huang, Y.J. Titanium oxide shell coatings decrease the cytotoxicity of zno nanoparticles. *Chemical research in toxicology* **2011**, *24*, 303-313.
9. Jugan, M.L.; Barillet, S.; Simon-Deckers, A.; Herlin-Boime, N.; Sauvaigo, S.; Douki, T.; Carriere, M. Titanium dioxide nanoparticles exhibit genotoxicity and impair DNA repair activity in a549 cells. *Nanotoxicology* **2012**, *6*, 501-513.
10. Kroll, A.; Dierker, C.; Rommel, C.; Hahn, D.; Wohlleben, W.; Schulze-Isfort, C.; Gobbert, C.; Voetz, M.; Hardinghaus, F.; Schnekenburger, J. Cytotoxicity screening of 23 engineered nanomaterials using a test matrix of ten cell lines and three different assays. *Particle and fibre toxicology* **2011**, *8*, 9.
11. Mano, S.S.; Kanehira, K.; Sonezaki, S.; Taniguchi, A. Effect of polyethylene glycol modification of tio2 nanoparticles on cytotoxicity and gene expressions in human cell lines. *International journal of molecular sciences* **2012**, *13*, 3703-3717.
12. Nemmar, A.; Melghit, K.; Al-Salam, S.; Zia, S.; Dhanasekaran, S.; Attoub, S.; Al-Amri, I.; Ali, B.H. Acute respiratory and systemic toxicity of pulmonary exposure to rutile fe-doped tio(2) nanorods. *Toxicology* **2011**, *279*, 167-175.
13. Panas, A.; Marquardt, C.; Nalcaci, O.; Bockhorn, H.; Baumann, W.; Paur, H.R.; Mulhopt, S.; Diabate, S.; Weiss, C. Screening of different metal oxide nanoparticles reveals selective toxicity

and inflammatory potential of silica nanoparticles in lung epithelial cells and macrophages. *Nanotoxicology* **2013**, *7*, 259-273.

14. Shi, Y.; Wang, F.; He, J.; Yadav, S.; Wang, H. Titanium dioxide nanoparticles cause apoptosis in beas-2b cells through the caspase 8/t-bid-independent mitochondrial pathway. *Toxicology letters* **2010**, *196*, 21-27.
15. Tedja, R.; Lim, M.; Amal, R.; Marquis, C. Effects of serum adsorption on cellular uptake profile and consequent impact of titanium dioxide nanoparticles on human lung cell lines. *ACS nano* **2012**, *6*, 4083-4093.
16. Val, S.; Hussain, S.; Boland, S.; Hamel, R.; Baeza-Squiban, A.; Marano, F. Carbon black and titanium dioxide nanoparticles induce pro-inflammatory responses in bronchial epithelial cells: Need for multiparametric evaluation due to adsorption artifacts. *Inhalation toxicology* **2009**, *21 Suppl 1*, 115-122.
17. Wan, R.; Mo, Y.; Feng, L.; Chien, S.; Tollerud, D.J.; Zhang, Q. DNA damage caused by metal nanoparticles: Involvement of oxidative stress and activation of atm. *Chemical research in toxicology* **2012**, *25*, 1402-1411.
18. Zarogiannis, S.G.; Filippidis, A.S.; Fernandez, S.; Jurkuvenaite, A.; Ambalavanan, N.; Stanishevsky, A.; Vohra, Y.K.; Matalon, S. Nano-tio(2) particles impair adhesion of airway epithelial cells to fibronectin. *Respir.Physiol Neurobiol.* **2013**, *185*, 454-460.
19. Andersson, P.O.; Lejon, C.; Ekstrand-Hammarstrom, B.; Akfur, C.; Ahlinder, L.; Bucht, A.; Osterlund, L. Polymorph- and size-dependent uptake and toxicity of tio(2) nanoparticles in living lung epithelial cells. *Small* **2011**, *7*, 514-523.
20. Muller, L.; Riediker, M.; Wick, P.; Mohr, M.; Gehr, P.; Rothen-Rutishauser, B. Oxidative stress and inflammation response after nanoparticle exposure: Differences between human lung cell monocultures and an advanced three-dimensional model of the human epithelial airways. *Journal of the Royal Society, Interface / the Royal Society* **2010**, *7 Suppl 1*, S27-40.
21. Rushton, E.K.; Jiang, J.; Leonard, S.S.; Eberly, S.; Castranova, V.; Biswas, P.; Elder, A.; Han, X.; Gelein, R.; Finkelstein, J., *et al.* Concept of assessing nanoparticle hazards considering nanoparticle dosemetric and chemical/biological response metrics. *Journal of toxicology and environmental health. Part A* **2010**, *73*, 445-461.
22. Toyooka, T.; Amano, T.; Ibuki, Y. Titanium dioxide particles phosphorylate histone h2ax independent of ros production. *Mutat.Res* **2012**, *742*, 84-91.
