# Supplementary material for: Collection of Controlled Nanosafety Data—The CoCoN-Database, a Tool to Assess Nanomaterial Hazard
Source: Nanomaterials (Basel). 2022 Jan 28;12(3):441. doi: 10.3390/nano12030441 (PMC8839907; doi:10.3390/nano12030441)
Supplement: Supplementary file 1 [file nanomaterials-12-00441-s001.zip › Supplementary Material Reference List in vivo Studies final2.pdf]

References

3.1. In vivo Studies

The data for figure 6 were extracted from the publications listed below.

3.1.1. Instillation Studies - [1-29]

3.1.2. Inhalation Studies - [4,11,20,30-52]

1. Ahn, M.H.; Kang, C.M.; Park, C.S.; Park, S.J.; Rhim, T.; Yoon, P.O.; Chang, H.S.; Kim, S.H.; Kyono, H.; Kim, K.C. Titanium dioxide particle-induced goblet cell hyperplasia: Association with mast cells and il-13. *Respiratory research* **2005**, *6*, 34.
2. Chen, H.W.; Su, S.F.; Chien, C.T.; Lin, W.H.; Yu, S.L.; Chou, C.C.; Chen, J.J.; Yang, P.C. Titanium dioxide nanoparticles induce emphysema-like lung injury in mice. *FASEB journal : official publication of the Federation of American Societies for Experimental Biology* **2006**, *20*, 2393-2395.
3. Cho, W.S.; Duffin, R.; Poland, C.A.; Howie, S.E.; MacNee, W.; Bradley, M.; Megson, I.L.; Donaldson, K. Metal oxide nanoparticles induce unique inflammatory footprints in the lung: Important implications for nanoparticle testing. *Environmental health perspectives* **2010**, *118*, 1699-1706.
4. Christensen, F.M.; Johnston, H.J.; Stone, V.; Aitken, R.J.; Hankin, S.; Peters, S.; Aschberger, K. Nano-tio(2)--feasibility and challenges for human health risk assessment based on open literature. *Nanotoxicology* **2011**, *5*, 110-124.
5. Han, X.; Corson, N.; Wade-Mercer, P.; Gelein, R.; Jiang, J.; Sahu, M.; Biswas, P.; Finkelstein, J.N.; Elder, A.; Oberdorster, G. Assessing the relevance of in vitro studies in nanotoxicology by examining correlations between in vitro and in vivo data. *Toxicology* **2012**, *297*, 1-9.
6. Ho, C.C.; Chang, H.; Tsai, H.T.; Tsai, M.H.; Yang, C.S.; Ling, Y.C.; Lin, P. Quantum dot 705, a cadmium-based nanoparticle, induces persistent inflammation and granuloma formation in the mouse lung. *Nanotoxicology* **2013**, *7*, 105-115.
7. Höhr, D.; Steinfartz, Y.; Schins, R.P.; Knaapen, A.M.; Martra, G.; Fubini, B.; Borm, P.J. The surface area rather than the surface coating determines the acute inflammatory response after instillation of fine and ultrafine tio2 in the rat. *Int.J.Hyg.Environ.Health* **2002**, *205*, 239-244.
8. Kobayashi, N.; Naya, M.; Endoh, S.; Maru, J.; Yamamoto, K.; Nakanishi, J. Comparative pulmonary toxicity study of nano-tio(2) particles of different sizes and agglomerations in rats: Different short- and long-term post-instillation results. *Toxicology* **2009**, *264*, 110-118.
9. Liang, G.; Pu, Y.; Yin, L.; Liu, R.; Ye, B.; Su, Y.; Li, Y. Influence of different sizes of titanium dioxide nanoparticles on hepatic and renal functions in rats with correlation to oxidative stress. *Journal of toxicology and environmental health. Part A* **2009**, *72*, 740-745.
10. Liu, H.; Yang, D.; Yang, H.; Zhang, H.; Zhang, W.; Fang, Y.; Lin, Z.; Tian, L.; Lin, B.; Yan, J., *et al.* Comparative study of respiratory tract immune toxicity induced by three sterilisation nanoparticles: Silver, zinc oxide and titanium dioxide. *Journal of hazardous materials* **2013**, *248-249*, 478-486.
11. Morimoto, Y.; Kobayashi, N.; Shinohara, N.; Myojo, T.; Tanaka, I.; Nakanishi, J. Hazard assessments of manufactured nanomaterials. *Journal of occupational health* **2010**, *52*, 325-334.
12. Naya, M.; Kobayashi, N.; Ema, M.; Kasamoto, S.; Fukumuro, M.; Takami, S.; Nakajima, M.; Hayashi, M.; Nakanishi, J. In vivo genotoxicity study of titanium dioxide nanoparticles using comet assay following intratracheal instillation in rats. *Regulatory toxicology and pharmacology : RTP* **2012**, *62*, 1-6.
13. Nemmar, A.; Melghit, K.; Al-Salam, S.; Zia, S.; Dhanasekaran, S.; Attoub, S.; Al-Amri, I.; Ali, B.H. Acute respiratory and systemic toxicity of pulmonary exposure to rutile fe-doped tio(2) nanorods. *Toxicology* **2011**, *279*, 167-175.
14. Park, E.J.; Shim, H.W.; Lee, G.H.; Kim, J.H.; Kim, D.W. Comparison of toxicity between the different-type tio(2) nanowires in vivo and in vitro. *Archives of toxicology* **2013**.
15. Park, E.J.; Yoon, J.; Choi, K.; Yi, J.; Park, K. Induction of chronic inflammation in mice treated with titanium dioxide nanoparticles by intratracheal instillation. *Toxicology* **2009**, *260*, 37-46.

16. Saber, A.T.; Jacobsen, N.R.; Mortensen, A.; Szarek, J.; Jackson, P.; Madsen, A.M.; Jensen, K.A.; Koponen, I.K.; Brunborg, G.; Gutzkow, K.B., *et al.* Nanotitanium dioxide toxicity in mouse lung is reduced in sanding dust from paint. *Particle and fibre toxicology* **2012**, *9*, 4.
17. Saber, A.T.; Koponen, I.K.; Jensen, K.A.; Jacobsen, N.R.; Mikkelsen, L.; Moller, P.; Loft, S.; Vogel, U.; Wallin, H. Inflammatory and genotoxic effects of sanding dust generated from nanoparticle-containing paints and lacquers. *Nanotoxicology* **2012**, *6*, 776-788.
18. Sager, T.M.; Castranova, V. Surface area of particle administered versus mass in determining the pulmonary toxicity of ultrafine and fine carbon black: Comparison to ultrafine titanium dioxide. *Particle and fibre toxicology* **2009**, *6*, 15.
19. Sager, T.M.; Kommineni, C.; Castranova, V. Pulmonary response to intratracheal instillation of ultrafine versus fine titanium dioxide: Role of particle surface area. *Particle and fibre toxicology* **2008**, *5*, 17.
20. Skocaj, M.; Filipic, M.; Petkovic, J.; Novak, S. Titanium dioxide in our everyday life; is it safe? *Radiology and oncology* **2011**, *45*, 227-247.
21. Wang, J.; Chen, C.; Liu, Y.; Jiao, F.; Li, W.; Lao, F.; Li, Y.; Li, B.; Ge, C.; Zhou, G., *et al.* Potential neurological lesion after nasal instillation of tio(2) nanoparticles in the anatase and rutile crystal phases. *Toxicology letters* **2008**, *183*, 72-80.
22. Wang, J.; Liu, Y.; Jiao, F.; Lao, F.; Li, W.; Gu, Y.; Li, Y.; Ge, C.; Zhou, G.; Li, B., *et al.* Time-dependent translocation and potential impairment on central nervous system by intranasally instilled tio(2) nanoparticles. *Toxicology* **2008**, *254*, 82-90.
23. Warheit, D.B.; Webb, T.R.; Reed, K.L.; Frerichs, S.; Sayes, C.M. Pulmonary toxicity study in rats with three forms of ultrafine-tio2 particles: Differential responses related to surface properties. *Toxicology* **2007**, *230*, 90-104.
24. Warheit, D.B.; Webb, T.R.; Sayes, C.M.; Colvin, V.L.; Reed, K.L. Pulmonary instillation studies with nanoscale tio2 rods and dots in rats: Toxicity is not dependent upon particle size and surface area. *Toxicological sciences : an official journal of the Society of Toxicology* **2006**, *91*, 227-236.
25. Yazdi, A.S.; Guarda, G.; Riteau, N.; Drexler, S.K.; Tardivel, A.; Couillin, I.; Tschopp, J. Nanoparticles activate the nlr pyrin domain containing 3 (nlrp3) inflammasome and cause pulmonary inflammation through release of il-1alpha and il-1beta. *Proceedings of the National Academy of Sciences of the United States of America* **2010**, *107*, 19449-19454.
26. Yokohira, M.; Hashimoto, N.; Yamakawa, K.; Suzuki, S.; Saoo, K.; Kuno, T.; Imaida, K. Lung carcinogenic bioassay of cuo and tio(2) nanoparticles with intratracheal instillation using f344 male rats. *Journal of toxicologic pathology* **2009**, *22*, 71-78.
27. Yokohira, M.; Kuno, T.; Yamakawa, K.; Hashimoto, N.; Ninomiya, F.; Suzuki, S.; Saoo, K.; Imaida, K. An intratracheal instillation bioassay system for detection of lung toxicity due to fine particles in f344 rats. *Journal of toxicologic pathology* **2009**, *22*, 1-10.
28. Yokohira, M.; Kuno, T.; Yamakawa, K.; Hosokawa, K.; Matsuda, Y.; Hashimoto, N.; Suzuki, S.; Saoo, K.; Imaida, K. Lung toxicity of 16 fine particles on intratracheal instillation in a bioassay model using f344 male rats. *Toxicologic pathology* **2008**, *36*, 620-631.
29. Zhang, L.; Bai, R.; Li, B.; Ge, C.; Du, J.; Liu, Y.; Le Guyader, L.; Zhao, Y.; Wu, Y.; He, S., *et al.* Rutile tio(2) particles exert size and surface coating dependent retention and lesions on the murine brain. *Toxicology letters* **2011**, *207*, 73-81.
30. Bermudez, E.; Mangum, J.B.; Asgharian, B.; Wong, B.A.; Reverdy, E.E.; Janszen, D.B.; Hext, P.M.; Warheit, D.B.; Everitt, J.I. Long-term pulmonary responses of three laboratory rodent species to subchronic inhalation of pigmentary titanium dioxide particles. *Toxicol.Sci.* **2002**, *70*, 86-97.
31. Bermudez, E.; Mangum, J.B.; Wong, B.A.; Asgharian, B.; Hext, P.M.; Warheit, D.B.; Everitt, J.I. Pulmonary responses of mice, rats, and hamsters to subchronic inhalation of ultrafine titanium dioxide particles. *Toxicol.Sci.* **2004**, *77*, 347-357.
32. Boisen, A.M.; Shipley, T.; Jackson, P.; Hougaard, K.S.; Wallin, H.; Yauk, C.L.; Vogel, U. Nanotio(2) (uv-titan) does not induce estr mutations in the germline of prenatally exposed female mice. *Particle and fibre toxicology* **2012**, *9*, 19.

33. Geiser, M.; Casaulta, M.; Kupferschmid, B.; Schulz, H.; Semmler-Behnke, M.; Kreyling, W. The role of macrophages in the clearance of inhaled ultrafine titanium dioxide particles. *American journal of respiratory cell and molecular biology* **2008**, *38*, 371-376.
34. Grassian, V.H.; O'Shaughnessy P, T.; Adamcakova-Dodd, A.; Pettibone, J.M.; Thorne, P.S. Inhalation exposure study of titanium dioxide nanoparticles with a primary particle size of 2 to 5 nm. *Environmental health perspectives* **2007**, *115*, 397-402.
35. Halappanavar, S.; Jackson, P.; Williams, A.; Jensen, K.A.; Hougaard, K.S.; Vogel, U.; Yauk, C.L.; Wallin, H. Pulmonary response to surface-coated nanotitanium dioxide particles includes induction of acute phase response genes, inflammatory cascades, and changes in micrornas: A toxicogenomic study. *Environmental and molecular mutagenesis* **2011**, *52*, 425-439.
36. Jackson, P.; Halappanavar, S.; Hougaard, K.S.; Williams, A.; Madsen, A.M.; Lamson, J.S.; Andersen, O.; Yauk, C.; Wallin, H.; Vogel, U. Maternal inhalation of surface-coated nanosized titanium dioxide (uv-titan) in c57bl/6 mice: Effects in prenatally exposed offspring on hepatic DNA damage and gene expression. *Nanotoxicology* **2013**, *7*, 85-96.
37. Kan, H.; Wu, Z.; Lin, Y.C.; Chen, T.H.; Cumpston, J.L.; Kashon, M.L.; Leonard, S.; Munson, A.E.; Castranova, V. The role of nodose ganglia in the regulation of cardiovascular function following pulmonary exposure to ultrafine titanium dioxide. *Nanotoxicology* **2014**, *8*, 447-454.
38. Kan, H.; Wu, Z.; Young, S.H.; Chen, T.H.; Cumpston, J.L.; Chen, F.; Kashon, M.L.; Castranova, V. Pulmonary exposure of rats to ultrafine titanium dioxide enhances cardiac protein phosphorylation and substance p synthesis in nodose ganglia. *Nanotoxicology* **2012**, *6*, 736-745.
39. Knuckles, T.L.; Yi, J.; Frazer, D.G.; Leonard, H.D.; Chen, B.T.; Castranova, V.; Nurkiewicz, T.R. Nanoparticle inhalation alters systemic arteriolar vasoreactivity through sympathetic and cyclooxygenase-mediated pathways. *Nanotoxicology* **2012**, *6*, 724-735.
40. Landsiedel, R.; Ma-Hock, L.; Kroll, A.; Hahn, D.; Schnekenburger, J.; Wiench, K.; Wohlleben, W. Testing metal-oxide nanomaterials for human safety. *Advanced materials* **2010**, *22*, 2601-2627.
41. LeBlanc, A.J.; Cumpston, J.L.; Chen, B.T.; Frazer, D.; Castranova, V.; Nurkiewicz, T.R. Nanoparticle inhalation impairs endothelium-dependent vasodilation in subepicardial arterioles. *Journal of toxicology and environmental health. Part A* **2009**, *72*, 1576-1584.
42. LeBlanc, A.J.; Moseley, A.M.; Chen, B.T.; Frazer, D.; Castranova, V.; Nurkiewicz, T.R. Nanoparticle inhalation impairs coronary microvascular reactivity via a local reactive oxygen species-dependent mechanism. *Cardiovascular toxicology* **2010**, *10*, 27-36.
43. Levy, L.; Chaudhuri, I.S.; Krueger, N.; McCunney, R.J. Does carbon black disaggregate in lung fluid? A critical assessment. *Chemical research in toxicology* **2012**, *25*, 2001-2006.
44. Lindberg, H.K.; Falck, G.C.; Catalan, J.; Koivisto, A.J.; Suhonen, S.; Jarventaus, H.; Rossi, E.M.; Nykasenoja, H.; Peltonen, Y.; Moreno, C., *et al.* Genotoxicity of inhaled nanosized tio(2) in mice. *Mutation research* **2012**, *745*, 58-64.
45. Morimoto, Y.; Oyabu, T.; Ogami, A.; Myojo, T.; Kuroda, E.; Hirohashi, M.; Shimada, M.; Lenggoro, W.; Okuyama, K.; Tanaka, I. Investigation of gene expression of mmp-2 and timp-2 mrna in rat lung in inhaled nickel oxide and titanium dioxide nanoparticles. *Industrial health* **2011**, *49*, 344-352.
46. Nurkiewicz, T.R.; Porter, D.W.; Hubbs, A.F.; Cumpston, J.L.; Chen, B.T.; Frazer, D.G.; Castranova, V. Nanoparticle inhalation augments particle-dependent systemic microvascular dysfunction. *Particle and fibre toxicology* **2008**, *5*, 1.
47. Nurkiewicz, T.R.; Porter, D.W.; Hubbs, A.F.; Stone, S.; Chen, B.T.; Frazer, D.G.; Boegehold, M.A.; Castranova, V. Pulmonary nanoparticle exposure disrupts systemic microvascular nitric oxide signaling. *Toxicological sciences : an official journal of the Society of Toxicology* **2009**, *110*, 191-203.
48. Oosthuizen, M.A.; Oberholzer, H.M.; Scriba, M.R.; van der Spuy, W.J.; Pretorius, E. Evaluation of the morphological changes in the lungs of balb/c mice after inhalation of spherical and rod-shaped titanium nanoparticles. *Micron* **2012**, *43*, 863-869.
49. Rossi, E.M.; Pylkkanen, L.; Koivisto, A.J.; Vippola, M.; Jensen, K.A.; Miettinen, M.; Sirola, K.; Nykasenoja, H.; Karisola, P.; Stjernvall, T., *et al.* Airway exposure to silica-coated tio2

nanoparticles induces pulmonary neutrophilia in mice. *Toxicological sciences : an official journal of the Society of Toxicology* **2010**, *113*, 422-433.
50. Scuri, M.; Chen, B.T.; Castranova, V.; Reynolds, J.S.; Johnson, V.J.; Samsell, L.; Walton, C.; Piedimonte, G. Effects of titanium dioxide nanoparticle exposure on neuroimmune responses in rat airways. *Journal of toxicology and environmental health. Part A* **2010**, *73*, 1353-1369.
51. van Ravenzwaay, B.; Landsiedel, R.; Fabian, E.; Burkhardt, S.; Strauss, V.; Ma-Hock, L. Comparing fate and effects of three particles of different surface properties: Nano-tio(2), pigmentary tio(2) and quartz. *Toxicology letters* **2009**, *186*, 152-159.
52. Warheit, D.B.; Donner, E.M. Rationale of genotoxicity testing of nanomaterials: Regulatory requirements and appropriateness of available oecd test guidelines. *Nanotoxicology* **2010**, *4*, 409-413.
