# Supplementary material for: Collection of Controlled Nanosafety Data—The CoCoN-Database, a Tool to Assess Nanomaterial Hazard
Source: Nanomaterials (Basel). 2022 Jan 28;12(3):441. doi: 10.3390/nano12030441 (PMC8839907; doi:10.3390/nano12030441)
Supplement: Supplementary file 1 [file Supplementary Material Table S1-2 final.pdf › Supplementary Material Table S1-2 final.pdf]

Table S1. Read out from the tabular form of the data collection on the effects of TiO_2 after lung exposure by instillation or aspiration.

Dataset	Type of TiO_2	Supplier / Size	Test Species	Exposure / Duration	Doses	Endpoint [mg/kg/d]	Basis	Ref.
Studies with Rats								
**1**	Aeroxide TiO_2 P25	Degussa Corporation (Parsippany, NJ) / 21 nm	Male Fisher CDF rats	Intratracheal instillation / 1 d; 7 d, 42 d	0.26, 0,52 and 1.04 mg/rat	LOAEL 0.26	TiO_2 induces at all concentrations an inflammatory response which decreases slowly over time	[1]
**2**	Anatase TiO_2	6-10 nm	Male Crl:CD(SD)IGS BR rats	Intratracheal instillation / 1 d, 7 d, 30 d, and 90 d	1 and 5 mg/kg	NOAEL after 7 d 5mg/kg	Both fine and ultrafine particles induce a transient increase in neutrophil migration after 24 h, which decreases rapidly after 7 d to control level. No real source given for the titanium dioxide and the animals.	[2]
**3**	Rutile TiO_2	300 nm	Male Crl:CD(SD)IGS BR rats	Intratracheal instillation / 1 d, 7 d, 30 d, and 90 d	1 and 5 mg/kg	NOAEL after 7 d 5mg/kg		
**4**	Anatase TiO_2	Millenium Chemical Corporation (Hunt Valley, MD) / 20 nm	Male Fischer 344 rats	Intratracheal instillation / 1 d	100 µg/animal	NOAEL 100	From the three variants of TiO_2 only the 25 nm sized material induced a detectable influx of neutrophils into the lungs. But this response is much lower compared to other particles made from silver or copper.	[3]
**5**	Anatase/Rutile TiO_2	Degussa Chemicals (Hanau, Germany) / 25 nm	Male Fischer 344 rats	Intratracheal instillation / 1 d	100 µg/animal	LOEL 100		
**6**	Anatase TiO_2	Fairlawn, NJ / 250 nm	Male Fischer 344 rats	Intratracheal instillation / 1 d	100 µg/animal	NOAEL 100		

**7**	TiO_2	30-40 nm	Female Wistar rats	Intratracheal instillation / 1 d	182 and 545 µg / animal	NOAEL 545	Supplier not given; Endotoxin-free materials, 24 h after the treatment no neutrophil influx into the lungs of the animals could be observed	[4]
**8**	Anatase TiO_2	Ishihara Sangyo Kaisha, Ltd., Osaka, Japan / 5 nm	Male Crl: CD rats	Single intratracheal instillation / 1 d	1 and 5 mg/kg	NOAEL 1	Material not tested for endotoxin. There is only a slight increase in neutrophils in BALF and no signs of genotoxicity at all treatments.	[5]
**9**	Anatase TiO_2	Ishihara Sangyo Kaisha, Ltd., Osaka, Japan / 5 nm	Male Crl: CD rats	Repeated intratracheal instillation / once a week for 5 weeks	0.2 and 1 mg/kg	NOAEL 0.2		
Studies with Mice								
**10**	Anatase TiO_2	Lolitec / 20 nm	C57BL/6J Mice and several knock-out variants	Intranasal instillation / 6 h	7.5 and 30 mg/kg	unclear which dose was instilled for data represented in Fig. 4	Recruitment of neutrophils into the BAL fluid could be demonstrated. As it is unclear which dose has been applied for the intranasal treatment shown in figure 4 and only 6 h have been observed, this study is of very low quality and reliability.	[6]
**11**	Rutile TiO_2	Lolitec / 80 nm	C57BL/6J Mice and several knock-out variants	Intranasal instillation / 6 h	7.5 and 30 mg/kg			
**12**	Anatase TiO_2	selfmade / 70 – 150 nm	Male C57BL/6J mice	Pharyngeal aspiration / 1, 3, 7, 28, and 112 d postexposure	2 and 30 µg/animal	NOAEL 30 µg	Up to seven days of observation for neutrophil influx no increase could be detected for the spherical particles	[7]

**13**	Anatase TiO_2	Sigma–Aldrich (Saint Quentin Fallavier, France) / 15 nm	Male BALB/c mice	Oropharyngeal aspiration / 1 - 2 d	0.8 mg/kg	NOAEL 0.8 (without pre-treatment)	Animals were dermally sensitized and after 14 days exposed to particles via aspiration. After 24 hours the animals were challenged with the sensitizer and 24 hours later the influx of neutrophils into the lungs was measured. Without challenge, no influx occurs, with the sensitisation an inflammatory response could be detected	[8]
**14**	Anatase/Rutile TiO_2	Degussa, Germany /19 nm	Female C57BL/6 mice	Intratracheal instillation / 1 d	54 µg/animal	LOEL 54	The nanoparticles only induced a statistically significant influx of immune cells into the lungs. The pure rutile particles only a small effect whereas the anatase particles induce a much higher level of neutrophils in the BALF	[9]
**15**	Rutile UV-Titan L181	Beck and Jorgensen, Denmark / 21 nm	Female C57BL/6 mice	Intratracheal instillation / 1 d	54 µg/animal	LOEL 54		
**16**	Rutile RDI-S, fine TiO_2	Kemira Pigments, Finland / 288 nm	Female C57BL/6 mice	Intratracheal instillation / 1 d	54 µg/animal	NOAEL 54		
**17**	Degussa P25	Nippon Aerosil Co. Ltd. Japan / 21.7 nm	ICR mice	Intratracheal instillation / 12, 17 and 90 days postexposure	500 µg/animal	NOAEL 500	All parameters were unchanged in BALF after the treatment with the particles.	[10]

Table S2. Read out from the tabular form of the data collection on the endpoint "inflammation/cell migration" induced by TiO_2 after lung exposure via inhalation.

Dataset	Type of TiO_2	Supplier / Size	Test Species	Exposure / Duration	Doses / Observation	Endpoint Inflammation	Basis	Ref.
Studies with Rats								
**1**	Nano TiO_2 Anatase/Rutile (79:30)	20 – 30 nm	Male Wistar rats	Inhalation / 6 h/d, 5 d	100 mg/m^3 / 3 and 14 days postexposure	Cell migration / neutrophil influx	Total cell count in BALF was increased	[11]
**2**	Pigmentary Rutile TiO_2	Kronos International / 200 nm	Male Wistar rats	Inhalation / 6 h/d, 5 d	250 mg/m^3 / 3 and 14 days postexposure	Cell migration / neutrophil influx	No granulocyte infiltration into the lungs took place	
**3**	Rutile TiO_2	Ishihara Industry / 35 nm	Male Wistar rats	Inhalation / 28 d	# 280´000/cm^3 6 h/day, 5 d/week, 4 weeks) / 4 d, 30 d and 90 d postexposure	Cell migration / neutrophil influx	Total cell count in BALF was not increased	[12]
**4**	Anatase TiO_2	Nanostructured and Amorphous Materials Inc. (Texas, USA) / 5 nm	Male Fisher 344 rats	Inhalation	2 and 7 mg/m^3 for 6 h / 16 h postexposure	Cell migration / neutrophil influx	No changes of BALF cell content and distribution at 2 mg/m^3. The higher dose provokes an increase of macrophages.	[13]
Studies with Mice								
**5**	Rutile TiO_2	Sigma Aldrich / 5 µm	BALB/c mice	Inhalation / 2 h/d	10 mg/m^3 / 24 h postexposure	Cell migration / neutrophil influx	Total cell count in BALF was not increased	[14]
**6**	Nano TiO_2 Anatase/Rutile	NanoAmor / 30 nm	BALB/c mice	Inhalation / 2 h/d	10 mg/m^3 / 24 h postexposure	Cell migration / neutrophil influx	Total cell count in BALF was not increased	

**7**	Anatase TiO_2	Sigma Aldrich / 25 nm	BALB/c mice	Inhalation / 2 h/d	10 mg/m^3 / 24 h postexposure	Cell migration / neutrophil influx	Total cell count in BALF was not increased	
**8**	in situ produced Anatase/Brookite TiO_2	21 nm	BALB/c mice	Inhalation / 2 h/d	10 mg/m^3 / 24 h postexposure	Cell migration / neutrophil influx	Total cell count in BALF was not increased	

1. Sager, T.M.; Castranova, V. Surface area of particle administered versus mass in determining the pulmonary toxicity of ultrafine and fine carbon black: Comparison to ultrafine titanium dioxide. *Particle and fibre toxicology* **2009**, *6*, 15.
2. Warheit, D.B.; Reed, K.L.; Sayes, C.M. A role for nanoparticle surface reactivity in facilitating pulmonary toxicity and development of a base set of hazard assays as a component of nanoparticle risk management. *Inhalation toxicology* **2009**, *21 Suppl 1*, 61-67.
3. Rushton, E.K.; Jiang, J.; Leonard, S.S.; Eberly, S.; Castranova, V.; Biswas, P.; Elder, A.; Han, X.; Gelein, R.; Finkelstein, J.*, et al.* Concept of assessing nanoparticle hazards considering nanoparticle dosemetric and chemical/biological response metrics. *J Toxicol Environ Health A* **2010**, *73*, 445-461.
4. Cho, W.S.; Duffin, R.; Poland, C.A.; Howie, S.E.; MacNee, W.; Bradley, M.; Megson, I.L.; Donaldson, K. Metal oxide nanoparticles induce unique inflammatory footprints in the lung: Important implications for nanoparticle testing. *Environmental health perspectives* **2010**, *118*, 1699-1706.
5. Naya, M.; Kobayashi, N.; Ema, M.; Kasamoto, S.; Fukumuro, M.; Takami, S.; Nakajima, M.; Hayashi, M.; Nakanishi, J. In vivo genotoxicity study of titanium dioxide nanoparticles using comet assay following intratracheal instillation in rats. *Regulatory toxicology and pharmacology : RTP* **2012**, *62*, 1-6.
6. Yazdi, A.S.; Guarda, G.; Riteau, N.; Drexler, S.K.; Tardivel, A.; Couillin, I.; Tschopp, J. Nanoparticles activate the nlr pyrin domain containing 3 (nlrp3) inflammasome and cause pulmonary inflammation through release of il-1alpha and il-1beta. *Proceedings of the National Academy of Sciences of the United States of America* **2010**, *107*, 19449-19454.
7. Porter, D.W.; Wu, N.; Hubbs, A.F.; Mercer, R.R.; Funk, K.; Meng, F.; Li, J.; Wolfarth, M.G.; Battelli, L.; Friend, S.*, et al.* Differential mouse pulmonary dose and time course responses to titanium dioxide nanospheres and nanobelts. *Toxicol Sci* **2013**, *131*, 179-193.
8. Hussain, S.; Vanoirbeek, J.A.; Luyts, K.; De Vooght, V.; Verbeken, E.; Thomassen, L.C.; Martens, J.A.; Dinsdale, D.; Boland, S.; Marano, F.*, et al.* Lung exposure to nanoparticles modulates an asthmatic response in a mouse model. *The European respiratory journal : official journal of the European Society for Clinical Respiratory Physiology* **2011**, *37*, 299-309.
9. Saber, A.T.; Jensen, K.A.; Jacobsen, N.R.; Birkedal, R.; Mikkelsen, L.; Moller, P.; Loft, S.; Wallin, H.; Vogel, U. Inflammatory and genotoxic effects of nanoparticles designed for inclusion in paints and lacquers. *Nanotoxicology* **2012**, *6*, 453-471.
10. Ho, C.C.; Chang, H.; Tsai, H.T.; Tsai, M.H.; Yang, C.S.; Ling, Y.C.; Lin, P. Quantum dot 705, a cadmium-based nanoparticle, induces persistent inflammation and granuloma formation in the mouse lung. *Nanotoxicology* **2013**, *7*, 105-115.
11. van Ravenzwaay, B.; Landsiedel, R.; Fabian, E.; Burkhardt, S.; Strauss, V.; Ma-Hock, L. Comparing fate and effects of three particles of different surface properties: Nano-tio(2), pigmentary tio(2) and quartz. *Toxicology letters* **2009**, *186*, 152-159.
12. Morimoto, Y.; Oyabu, T.; Ogami, A.; Myojo, T.; Kuroda, E.; Hirohashi, M.; Shimada, M.; Lenggoro, W.; Okuyama, K.; Tanaka, I. Investigation of gene expression of mmp-2 and timp-2 mrna in rat lung in inhaled nickel oxide and titanium dioxide nanoparticles. *Ind Health* **2011**, *49*, 344-352.
13. Noel, A.; Maghni, K.; Cloutier, Y.; Dion, C.; Wilkinson, K.J.; Halle, S.; Tardif, R.; Truchon, G. Effects of inhaled nano-tio2 aerosols showing two distinct agglomeration states on rat lungs. *Toxicology letters* **2012**, *214*, 109-119.

14. Rossi, E.M.; Pylkkanen, L.; Koivisto, A.J.; Vippola, M.; Jensen, K.A.; Miettinen, M.; Sirola, K.; Nykasenoja, H.; Karisola, P.; Stjernvall, T.*, et al.* Airway exposure to silica-coated tio2 nanoparticles induces pulmonary neutrophilia in mice. *Toxicological sciences : an official journal of the Society of Toxicology* **2010**, *113*, 422-433.
